# MolBioLib: a C++11 framework for rapid development and deployment of bioinformatics tasks

**DOI:** 10.1093/bioinformatics/bts458

**Published:** 2012-07-19

**Authors:** Toshiro K. Ohsumi, Mark L. Borowsky

**Affiliations:** ^1^Department of Molecular Biology, Massachusetts General Hospital, Richard B. Simches Research Center, 185 Cambridge Street, CPZN-7250 Boston, MA 02114 and ^2^Department of Genetics, Harvard Medical School, Boston, MA 02115, USA

## Abstract

**Summary:** We developed MolBioLib to address the need for adaptable next-generation sequencing analysis tools. The result is a compact, portable and extensively tested C++11 software framework and set of applications tailored to the demands of next-generation sequencing data and applicable to many other applications. MolBioLib is designed to work with common file formats and data types used both in genomic analysis and general data analysis. A central relational-database-like Table class is a flexible and powerful object to intuitively represent and work with a wide variety of tabular datasets, ranging from alignment data to annotations. MolBioLib has been used to identify causative single-nucleotide polymorphisms in whole genome sequencing, detect balanced chromosomal rearrangements and compute enrichment of messenger RNAs (mRNAs) on microtubules, typically requiring applications of under 200 lines of code. MolBioLib includes programs to perform a wide variety of analysis tasks, such as computing read coverage, annotating genomic intervals and novel peak calling with a wavelet algorithm. Although MolBioLib was designed primarily for bioinformatics purposes, much of its functionality is applicable to a wide range of problems. Complete documentation and an extensive automated test suite are provided.

**Availability:** MolBioLib is available for download at: http://sourceforge.net/projects/molbiolib

**Contact**: ohsumit@molbio.mgh.harvard.edu

## 1 INTRODUCTION

Next-generation sequencing requires a data analysis approach capable of handling large, complex and varied datasets, from large sets of reads to complex polymorphisms to existing feature files. In addition, the competitive nature of research demands rapid development of methods that are flexible enough to integrate new and quickly evolving algorithms. Tools have been developed to address these needs, such as GATK (McKenna *et al.*, 2010). However, packages written in Java (e.g. GATK) require the maximum memory heap space to be specified at run time (Oracle, 2011), limiting how the input data are formatted and handled. For example, a coverage program would require more memory to compute coverage of a query-ordered SAM file versus a position-ordered SAM file, because a sliding window of coverage cannot be used. Programs written in C++ do not require the heap size to be specified and are only limited by the amount of available memory. Other packages written in C++ have their strengths, but they also have limitations that suggest a niche for our software, MolBioLib. Arachne (Batzoglou *et al.*, 2002; Jaffe *et al.*, 2003), the .NET Bio project by Outercurve Foundation (Outercurve, 2012) and NCBI's C++ Toolkit (Vatakov, 2012) provide many functions, but are not compact and do not always clearly identify the primary objects. Furthermore, the .NET Bio project is specific to the Windows environment (Mercer, 2012) and Arachne is specific to a particular Linux environment. IBM's GenomicTools (Tsirigos, 2012) has many very useful tools, but addresses common bioinformatics tasks at a lower level than MolBioLib, such as providing command-line tools rather than a unified program to generate ChIP-seq output. Other packages, such as Bio++ (Dutheil, 2008), libsequence (Thornton, 2003) and TIGR++ (Majoros, 2012), are targeted toward specific applications and not designed to provide breadth of functionality. The package that most closely resembles MolBioLib's philosophy is SeqAn (Döring, 2008), though it is written in an older version of C++ and thus does not take advantage of the variadic templates or other modern features of C++11 (ISO/IEC, 2011).

MolBioLib fills the need for a platform-independent, extensively tested, compact and efficient C++11 library and an extensive set of bioinformatics applications that can be used to analyze data and rapidly develop new tools. MolBioLib's library includes a variety of useful objects and functions, such as a relational-database-like object, a text file reader object that simplifies data input, statistical functions and peak calling methods that can operate on any array of values, such as per base sequence coverage. In addition, MolBioLib includes a broad range of tools, such as to generate coverage, hits of reads to features and ChIP-seq, all in one unified package.

The design of MolBioLib is based on four principles. The first is to simplify bioinformatics programming in C++11, achieved by developing a library that includes many common bioinformatics tasks. For example, C++11 requires programmers to write specialized data structures to sort associated data keeping them together, such as feature information associated with a position. Additionally, to iterate either sequentially or randomly through a tab-separated-values (TSV) file and select values from specific columns would require the creation of a function to split a line on tabs and constructs to index and traverse a text file. These, and many other common tasks, are built into MolBioLib, thus greatly simplifying the code one needs to write. It is hoped that MolBioLib will allow bioinformaticians to consider C++11 as a possible language of choice. Second, MolBioLib is efficient. C++11 is used because it is the new standard that introduces constructs for making objects such as Table. C++11 is efficient since it is a compiled language with no inherent restriction on memory heap size at run time. Templates are used extensively to compact code, avoid inefficient virtual table lookups and maintain type safety. Objects and method parameters are often templated so that they may be in-lined by the compiler. Third, MolBioLib promotes clarity and compactness by consolidating common operations into a concise set of objects. We also provide an extensive library of functions that are not intrinsic to one object, such as those that convert one data type to another, e.g. splitString converts a string to a vector<string>.

Given the range of problems MolBioLib addresses, the source code is compact: ~10 000 lines of code and comments for the core objects and functions. Among the 101 included applications, 86% are coded in fewer than 200 lines and 59% in fewer than 100 lines. In contrast, without such a framework, the user would have to code the thousands of lines of code to reproduce MolBioLib's functionality. Finally, MolBioLib is extensively tested and facilitates easy testing and debugging of its applications. Automated tests are provided for all objects and functions. Additional validation of the code base comes from extensive application of MolBioLib to many molecular biology projects (Lau *et al.*, 2009; Sharp *et al.*, 2011; Talkowski *et al.*, 2011; Zhao *et al.*, 2010; Raif S. Geha, manuscript in preparation). To simplify use of MolBioLib, all libraries are include files following the Boost convention (Schaling, 2011). Debugging and memory checking is thus facilitated with tools such as with Valgrind (Nethercote and Seward, 2007; Seward and Nethercote, 2005) since applications in MolBioLib consist of a main program file with many include files. Additional input and programming checks are incorporated into the framework through optional compiler flags.

## 2 METHODS

MolBioLib is hierarchically structured for ease of use. It contains three main components: the library consisting of a set of objects and functions, the set of applications and the documentation.


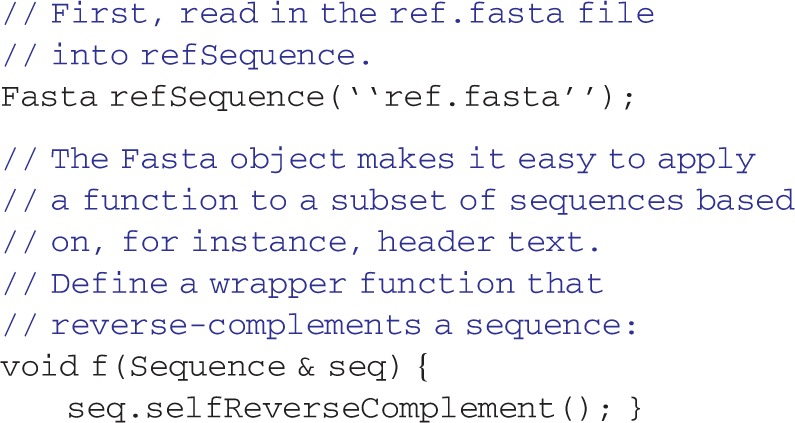


A script is included to compile all the external packages [such as BAMTools (Barnett *et al.*, 2011)], applications and optionally build and run tests. MolBioLib can be used independently of the external packages and interfaces. The documentation for all of MolBioLib may be generated automatically using the included Doxygen configuration file (van Heesch, 2011). The introductory pages of the Doxygen output show how to compile and use MolBioLib both as a set of tools as well as a programming framework. Functions that transform one data type to another are separated from the objects. Finally, the applications are hierarchically organized by usage type.


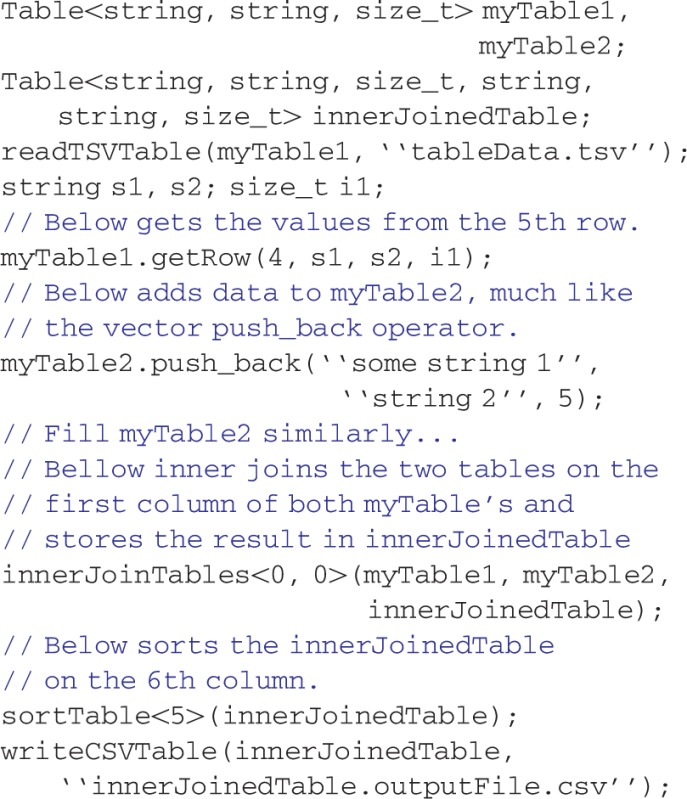


Several novel classes power rapid development with MolBioLib. The primary object that stores data in MolBioLib is Table, which is a container class similar to the C++ STL vector class except that each column may store a different data type, through C++11's variadic templates. Variadic templates allow the coding of objects that can accept an arbitrary number of template parameters. However, writing variadic templated objects and functions can become cumbersome (Gregor and Järvi, 2008; Kalev, 2008). Therefore, MolBioLib includes the Table object to represent tabular data, a mainstay of bioinformatics data exchange, in an intuitive and easily used fashion. The Table structure was based on the relational database (Codd, 1970) model, where related data are stored row-wise. Column data types are specified through the template parameters. A Table may be thought of as a generalized vector. It includes data row insertion and retrievals operations that are simple to use. Database-like operations for Table, such as concatenation, filtering and inner and outer joins, are provided. Example usage of a Table is:

where the readTSVTable is a function to read a TSV file into a table. This tabular grouping of data can be used for many bioinformatics tasks. One example is the Fasta object, derived from the Table object, which stores sequences and their headers. The Fasta object simplifies access to sequence data:

The primary object to read text files is ReadOnlyStringFile. The class automatically creates an index of a file, if not already present, so the file may be accessed as if it were a string array. The index file is created by going through the text file once and noting the starting file position of each line in the index file. The index file itself has a fixed length per line, simplifying the process of finding the index position. Thus, to access a line in the text file, the appropriate line in the index file is looked up. Subsequently, the line starting at the file position indicated by the look up is read in. Almost no memory is required in using the index. A ReadOnlyStringFile object functions like an array in which each element is one row of the file. ReadOnlyTSVFile is a particularly useful derived class of ReadOnlyStringFile. The values of each tab-separated field of each row can be accessed by the operator[] method, returning a vector containing the parsed elements of one row. Applications written with MolBioLib capitalize on the ability of the ReadOnlyStringFile class to hide all the housekeeping chores involved in parsing data from delimited text files.

For example, to sample a random subset of a TSV input file, one would code:

If a file only needs to be traversed once, sequentially a line at a time, ReadOnlyStringFile can traverse the file without creating an index file. This eliminates the time to build and store an index.

The ReadOnlySequencesFile, based on the ReadOnlyStringFile, is a FASTA/FASTQ reader object. It can work in a random access mode or sequentially traverse the file, providing all the read-only operations of the Fasta object, thus greatly simplifying access to sequence read files.


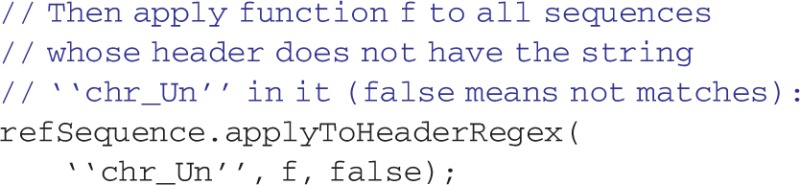


Other general objects in MolBioLib include a sparse vector object, e.g. for use in storing sparse coverage, a map facilitator between keys and rows in a Table, and a parameterized type interval object with associated overlap and set functions. A random number generator class that includes a permutation vector is also provided. Other bioinformatics objects included are an alignment object, a Sequence class with operations such as reverse-complementing, a feature object and a peak object for storing local extrema of numeric data. All of these classes have been used to simplify coding of novel bioinformatics analyses.


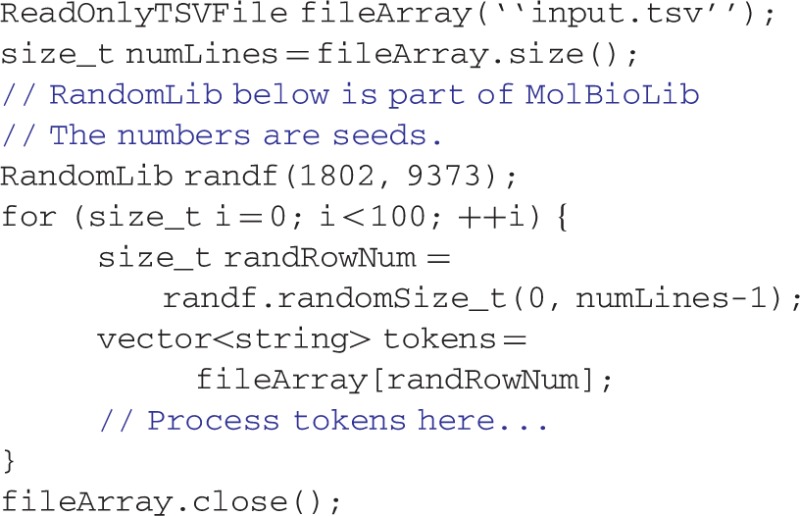


Functions included in MolBioLib are diverse, intended to cover four broad categories: algorithms, file readers and writers for various data types, system utilities and transformation of data types to different data types. Algorithm functions include Table functions, peak detection and statistics. Table functions include smoothing values, sorting and upper_bound and lower_bound of Tables analogous to their vector class counterparts. File reader and writers include those for various alignment formats, including SAM (Li *et al.*, 2009), Helicos BioSciences' TXT format (Helicos, 2010), NCBI tabular BLAST output (Madden, 2003), feature readers for tabular files such as for UCSC's refGene annotation table (refGene.txt; Kent, 2012), Ensembl's Biomart in TSV format (Flicek *et al.*, 2011; Smedley *et al.*, 2009) and the GFF format (Wellcome, 2012), streams for intervals and vectors, tuple streams and writers and Table reader and writers. Peak detection functions for numeric arrays will be discussed in more detail in the ChIP-seq section.

System utilities include a powerful command-line parsing system. Various command-line argument types are provided, including numeric and string. Input and output file name argument types provide file checking to ensure all input files are present at run time and to prevent accidental overwrites of output files. Furthermore, the command-line parser automatically records the date and time the program was compiled as well as the command line to simplify documentation of computational steps and pipelines. System utilities also provide functions to transform one data type to another, such as string conversions to and from various data types as well as string splitting (on one or more delimiters).

The compiler of choice for MolBioLib is clang++ version 3.0 and above (Clang, 2012) using the associated libc++ library and is available on the Linux, Mac OS X and MS Windows platforms. It supports a large subset of C++11, has very good compiler error messages and is efficient. MolBioLib also works with GNU g++ 4.7 and above (Gcc, 2012).

## 3 RESULTS

One of the primary goals of MolBioLib is to provide a set of programs that address the most common bioinformatics analyses. Here we describe applications in MolBioLib that address four common bioinformatics analyses: annotating a list of features, counts of hits to features, coverage and ChIP-Seq. We also touch upon additional useful utilities included in MolBioLib.

The MolBioLib application addFeaturesToTSVFile performs the very common task of adding gene annotations or more generically ‘features’, to an input file in which each row describes a genomic interval. Examples of annotations include the refGene.txt file downloadable from UCSC's genome browser site (Fujita *et al.*, 2011) that contains the gene ID, name, chromosome, strand, and start and stop positions of the transcript. Other annotation files include tabular data from the Ensembl/Biomart website (Flicek *et al.*, 2011; Smedley *et al.*, 2009), where one can download any set of genes with user-selected attributes such as IDs, names, positions, expression data and protein domain. There are numerous other annotation sources, many of which consist of carefully curated private data, on a topical website, or in a published supplement to a journal article. Other common tabular formats include the BED, PSL and GFF formats (Kent, 2012; Wellcome, 2012). Using addFeaturesToTSVFile, the genomic footprint of any such annotation can be intersected with another TSV file containing genomic intervals. The application will use the genomic interval specified on each row of the input file and find all intersecting feature coordinates (with matching strand, if specified) and add the appropriate annotation(s) to the row in the output. Importantly, this application will take in any input TSV as well as any annotations in TSV form (such as those noted above) and thus may be used on a wide variety of projects.

Another common bioinformatics task is to count the number of alignments mapping to a set of features in a TSV file, such as refGene.txt, promoter regions or classes of sequence repeats. For example, we have used this method to quantify the number of reads derived from genic regions, different classes of genomic repeats and from different classes of non-coding RNAs (Sharp *et al.*, 2011). refFeaturesAnalysis offers a number of options, such as shifting the positions of features (e.g. probing hits to upstream UTRs instead of the genes), filtering for a specific set of reads, maximum error in alignments and RPKM normalization (Mortazavi *et al.*, 2008). The input features are printed along with the count of alignments overlapping each input feature and meeting any filtering criteria.

Sequence coverage can be used for determining the number of reads mapped to a base or region and also for finding polymorphisms. MolBioLib has a unique coverage tool that outputs strand-specific statistics as well as a count of mismatched bases observed at each position. Coverage can be run on a user-defined set of regions and normalized to the number of million reads in the input reads file. Additionally, coverage can be executed using only the midpoint position to identify where the reads align. Finally, coverage can be run using uniquely aligning reads. Programs are provided to post-process coverage output in various ways, such as multiplying by some constants (either for the whole file or by contig/chromosome), windowing the coverage and converting the coverage to a wiggle file. Coverage output is the input for the ChIP-Seq analysis program discussed next.

An experimental ChIP-Seq program with a novel wavelet method is our final example of application of the MolBioLib library. Peak detection is one major next-generation sequencing application. Given an alignment file, this algorithm finds the coverage on each strand at each location, computed either per base or in bins spanning a user-defined number of bases. This is done for both the sample as well as for a background sample or control, and coverage is smoothed using a kernel smoother. One challenge of peak detection is finding peaks having a wide range of widths and heights. We address this by applying a translation-independent wavelet smoother applied at various scales, finding local peaks at each scale, and then ranking putative peaks by using ridge lines that identify peaks detected across multiple scales (Du *et al.*, 2006). Peaks with a longer ridge length are more isolated from other peaks, because they show up as peaks at various length scales. Optional filters remove low signals and spikes. This peak detection method is included in MolBioLib. Other smoothers, such as Gaussian, are also implemented in MolBioLib.

We validated the wavelet peak detector on published H3K4me3 ChIP-Seq data (Myers *et al.*, 2011). As expected, H3K4me3 peaks are enriched in promoter regions (Zhao *et al.*, 2007). The percentage of peaks in promoter regions was 57% compared to 33% using the window tag density method (Kharchenko *et al.*, 2008) and 53% using MACS (Zhang *et al.*, 2008). Peaks were well defined, with a mean width of 232 bp compared to 2840 bp and 654 bp for the other two methods tested on the same data.

There are many other applications included with MolBioLib that address much of the essential bioinformatics analyses done in next-generation sequencing projects. Some of the more common tasks include computing statistics on a list of numbers, creating histogram files from data files (both numeric and string), converting alignment formats. Additional tasks include common operations on FASTA and FASTQ files, such as obtaining a subset, trimming and removing duplicate reads. Moreover, there are programs to combine, print subsets and inner join TSV files. Intersection, subtraction and union operations of text files are also included.

## 4 DISCUSSION

MolBioLib fills the need for an efficient, reliable and compact C++11 bioinformatics framework. It is portable across many platforms and aligner formats and is fully documented. MolBioLib is unique in offering complete analysis programs for a variety of other very common tasks not addressed by other toolkits, from feature hit counts to coverage to ChIP-Seq.

MolBioLib classes offer considerable power and convenience for creating novel analysis applications. A central and very general Table class simulating the functionality of a database eases construction of many programs. The Table class is based on a collection vectors, thus having a small memory overhead compared to other data structures such as a map. Capacity for larger datasets is only limited by the amount of available memory. File readers provide efficient methods to perform ubiquitous file I/O tasks. These classes will have general utility for application development beyond the specific needs of computational biology.

As MolBioLib gains adoption, we aim to incorporate many of the applications both user-contributed and those developed for our projects into the main distribution through the SourceForge.net code repository mechanism.

## References

[bts458-B1] Barnett DW (2011). BamTools: a C++ API and toolkit for analyzing and managing BAM files. Bioinformatics.

[bts458-B2] Batzoglou S (2002). ARACHNE: a whole-genome shotgun assembler. Genome Res..

[bts458-B3] Clang (2012).

[bts458-B4] Codd EF (1970). A relational model of data for large shared data banks. Commun. ACM.

[bts458-B5] Döring A (2008). SeqAn—an efficient, generic C++ library for sequence analysis. BMC Bioinfromatics.

[bts458-B6] Du P (2006). Improved peak detection in mass spectrum by incorporating continuous wavelet transform-based pattern matching. Bioinformatics.

[bts458-B7] Dutheil J, Boussau B (2008). Non-homogeneous models of sequence evolution in the Bio++ suite of libraries and programs. BMC Evol. Biol..

[bts458-B8] Flicek P (2011). Ensembl 2011. Nucleic Acids Res..

[bts458-B9] Fujita PA (2011). The UCSC Genome Browser database: update 2011. Nucleic Acids Res..

[bts458-B10] Gcc (2012).

[bts458-B11] Gregor D, Järvi J (2008). Variadic Templates for C++0x. Special Issue OOPS Track at SAC 2007.

[bts458-B12] Helicos (2010). Helisphere User's Guide.

[bts458-B13] ISO/IEC (2011). ISO/IEC 14882:2011. Programming Languages C++.

[bts458-B14] Jaffe DB (2003). Whole-genome sequence assembly for mammalian genomes: Arachne 2. Genome Res..

[bts458-B15] Kalev D (2008). http://www.informit.com/guides/content.aspx?g=cplusplus&seqNum=399.

[bts458-B16] Karolchik D, Hinrichs AS, Furey TS, Roskin KM, Sugnet CW, Haussler D, Kent WJ (2004). The UCSC Table Browser data retrieval tool. Nucleic Acids Res..

[bts458-B17] Kharchenko PV (2008). Design and analysis of ChIP-seq experiments for DNA-binding proteins. Nat. Biotechnol..

[bts458-B18] Lau NC (2009). Systematic and single cell analysis of Xenopus Piwi-interacting RNAs and Xiwi. EMBO J..

[bts458-B19] Li H (2009). The sequence alignment/map format and SAMtools. Bioinformatics.

[bts458-B20] Madden T, McEntyre J, Ostell J (2003). The BLAST sequence analysis tool. The NCBI Handbook.

[bts458-B21] Majoros B (2012). www.cbcb.umd.edu/software/pirate/tigr++.shtml.

[bts458-B22] McKenna A (2010). The genome analysis toolkit: a MapReduce framework for analyzing next-generation DNA sequencing data. Genome Res..

[bts458-B23] Mercer SJ (2012).

[bts458-B24] Mortazavi A (2008). Mapping and quantifying mammalian transcriptomes by RNA-Seq. Nat. Methods.

[bts458-B25] Myers RM (2011). A user's guide to the encyclopedia of DNA elements (ENCODE). PLoS Biol..

[bts458-B26] Nethercote N, Seward J (2007). Valgrind: a framework for heavyweight dynamic binary instrumentation. ACM SIGPLAN 2007 Conference on Programming Language Design and Implementation (PLDI 2007).

[bts458-B27] Oracle (2011). http://docs.oracle.com/javase/6/docs/technotes/tools/solaris/java.html.

[bts458-B28] Outercurve (2012).

[bts458-B29] Schaling B (2011). The Boost C++ Libraries.

[bts458-B30] Seward J, Nethercote N (2005). Using Valgrind to detect undefined value errors with bit-precision. UNSENIX '05 Annual Technical Conference.

[bts458-B31] Sharp JA (2011). Functional analysis of the microtubule-interacting transcriptome. Mol. Biol. Cell.

[bts458-B32] Smedley D (2009). BioMart—biological queries made easy. BMC Genom..

[bts458-B33] Talkowski ME (2011). Next-generation sequencing strategies enable routine detection of balanced chromosome rearrangements for clinical diagnostics and genetic research. Am. J. Hum. Genet..

[bts458-B34] Thornton K (2003). libsequence: a C++ class library for evolutionary genetic analysis. Bioinformatics.

[bts458-B35] Tsirigos A (2012). GenomicTools: a computational platform for developing high-throughput analytics in genomics. Bioinformatics.

[bts458-B36] van Heesch D (2011). http://www.stack.nl/~dimitri/doxygen/index.html.

[bts458-B37] Vatakov D (2012). www.ncbi.nlm.nih.gov/IEB/ToolBox/CPP_DOC.

[bts458-B38] Wellcome (2012). GFF. GFF: an Exchange Format for Feature Description.

[bts458-B39] Zhang Y (2008). Model-based analysis of ChIP-Seq (MACS). Genome Biol..

[bts458-B40] Zhao J (2010). Genome-wide identification of polycomb-associated RNAs by RIP-seq. Mol. Cell.

[bts458-B41] Zhao XD (2007). Whole-genome mapping of histone H3 Lys4 and 27 trimethylations reveals distinct genomic compartments in human embryonic stem cells. Cell Stem Cell.

